# Genomic Analysis of a New Serovar of *Leptospira weilii* Serogroup Manhao

**DOI:** 10.3389/fmicb.2017.00149

**Published:** 2017-02-02

**Authors:** Yinghua Xu, Huajun Zheng, Ying Zhang, Yuezhu Wang, Jinlong Zhang, Zhe Li, Shenghui Cui, Xiaofang Xin, Qiang Ye, Yung-Fu Chang, Junzhi Wang

**Affiliations:** ^1^Key Laboratory of the Ministry of Health for Research on Quality and Standardization of Biotech Products, National Institutes for Food and Drug ControlBeijing, China; ^2^Key Laboratory of Reproduction Regulation of NPFPC, Shanghai Institute of Planned Parenthood Research, IRD, Fudan UniversityShanghai, China; ^3^Shanghai-MOST Key Laboratory of Health and Disease Genomics, Chinese National Human Genome Center at Shanghai, Zhangjiang Hi-Tech ParkShanghai, China; ^4^Department of Population Medicine and Diagnostic Sciences, Cornell University, IthacaNY, USA

**Keywords:** Leptospirosis, serogroup Manhao, new serovar, pathogenesis, genome

## Abstract

Leptospirosis, caused by pathogenic *Leptospira* spp., is recognized as an important emerging zoonotic disease throughout the world. In this study, multiple approaches were used to characterize the recently discovered serovar Heyan strain L231. This strain can infect guinea pigs and belonged to the pathogenic species *L. weilii*. Genome sequencing analysis revealed the draft genome of 4.2 M bp with a G+C content of 40.67% for strain L231, and a total of 4,794 ORFs were identified. The strain L231 genome was found to have a larger LPS biosynthesis locus than that of strains *L. interrogans* serovar Lai and *L. borgpetersenii* serovar Hardjobovis. Phylogenomic reconstructions showed that the evolutionary position of *L. weilii* serovar Heyan was different from that of other serovars from serogroup Manhao. These findings may lead us to a better understanding of *Leptospira* pathogenesis and evolution.

## Introduction

Leptospirosis, caused by pathogenic species of *Leptospira*, is recognized as an emerging zoonotic disease of global importance (Bharti et al., 2003). Humans are infected through contact with animal reservoirs or contaminated environment (i.e., soil, sewage, or water). It is estimated that there are approximately 1.03 million global leptospirosis cases each year, resulting in 58,900 deaths. In China, the earliest leptospirosis case was recorded in 1920s. So far, more than 2.5 million cases and over 20,000 deaths have been reported. In the past 60 years, 10 outbreaks of leptospirosis with incidence of more than 10 cases per 100,000 have occurred in China (Shi and Jiang, 2000).

To develop better control strategies for infectious diseases, such as new vaccines and diagnostic tests, further characterization of each pathogen is required. *Leptospira* strains are identified by serogroup, and the serogroups are further classified into different serovars using a conventional serological classification system (Kmety and Dikken, 1993). Although serogroups have no taxonomic value, they are useful for clinical and epidemiological studies (Levett, 2001). Until now, more than 250 pathogenic serovars belonging to 24 serogroups have been reported. Due to the variety of geographic and climatic conditions in China, pathogenic leptospiral serovar diversity is far greater than that of other countries, and more than 70 serovars arranged in 18 serogroups of pathogenic *Leptospira* from a variety of hosts have been isolated and identified and some of them (i.e., Manhao) are only found in China (Kmety and Dikken, 1993).

Recently, a new serovar (Heyan) of serogroup Manhao from a patient in Yunnan, China has been reported (Qin et al., 2004). The development of new molecular techniques has allowed for classification of the genus *Leptospira* into species, which may provide a better way of categorizing pathogenic *Leptospira* strains than that of traditional serological methods. Based on the classical DNA-DNA hybridization studies and 16S rRNA gene phylogeny, pathogenic *Leptospira* are now classified into nine species, *L. interrogans*, *L. borgpetersenii*, *L. kirschneri*, *L. alexanderi*, *L. alstonii, L. kmetyi*, *L. noguchii*, *L. santarosai*, and *L. weilii* (Brenner et al., 1999; Morey et al., 2006). A novel pathogenic species, designated *L. mayottensis* has recently been reported (Bourhy et al., 2014). Furthermore, pulsed-field gel electrophoresis, multiple-loci variable number tandem repeat analysis, and multi-locus sequence typing have also been developed for typing pathogenic *Leptospira* strains (Herrmann et al., 1992; Salaun et al., 2006; Xu et al., 2015; Zhang et al., 2015). More importantly, genome sequencing has been also used to analyze several *Leptospira* species and a high-level plasticity among pathogenic *Leptospira* genomes was revealed, which has improved our understanding of genetic diversity at the genome level (Ren et al., 2003; Bulach et al., 2006; Fouts et al., 2016; Xu et al., 2016). To further understand the molecular features and origin of those unique serogroups in China, multiple molecular approaches were used to characterize new *Leptospira* serovar Heyan in this study. In addition, the virulence of this new serovar was evaluated in the guinea pig challenge model.

## Materials and Methods

### *Leptospira* Strains and Culture

Serovar Heyan strain L231 was originally isolated from a clinical patient in Yunnan, China (Qin et al., 2004). *L. interrogans* serogroup Icterohaemorrhagiae serovar Icterohaemorrhagiae strain Lai was obtained from the collection maintained by the National Reference Laboratory for *Leptospira*, at the National Institutes of Food and Drug Control (NIFDC), Beijing, China (Qin, 1999). All leptospiral strains were cultivated at 28°C on modified TEPCKNN medium (10 mM Na_2_HPO4, 4 mM KH_2_PO4, pH 7.2; NIFDC, Beijing) supplemented with 10% rabbit serum. Genomic DNA was extracted using a Wizard Genomic DNA Purification Kit (Promega, Southampton, UK) following the manufacturer’s instructions. The extracted DNA was stored at -70°C until used.

### Virulence Testing and Histopathology

The animal experiments were authorized by the Animal Ethic Committee of NIFDC (License No. 2014-B-003), and conducted in compliance with Regulations on Management of Laboratory Animal (Ministry of Science and Technology) 1988, China.

The virulence of the isolate was evaluated using a guinea pig challenge model as previously described in the Chinese Pharmacopeia with minor modifications (Npc, 2010). Briefly, three Hartley female guinea pigs (180–200 g, from NIFDC), were inoculated intraperitoneally with 1 mL of leptospiral strain L231 containing 1 × 10^9^ organisms. Animals were injected with the same concentration of *L. interrogans* serovar Icterohaemorrhagiae strain Lai as a positive control while three guinea pigs were administrated with modified TEPCKNN medium as a negative control. Clinical symptoms were monitored by daily observations during the study period and moribund animals were euthanized immediately. Kidneys, lungs, and liver were collected and stored in 10% formalin for histopathological examination.

### 16S rRNA Analysis

16S rRNA analysis of strain L231 was performed as described previously (Merien et al., 1992). Briefly, the 16s rRNA gene was amplified with the primers LA (5′-GGCGGCGCGTCTT AAACATG-3′) and LB (5′-TTCCCCCCATTGAGCAAGATT-3′). The assembled sequence was then aligned against other 16S rRNA sequences available in the GenBank using BLAST^1^.

### Genome Sequencing and Assembly

Genome sequencing of strain L231 was performed using Illumina Hiseq 2500 (Illumina, Little Chesterford, Essex). The Illumina data were *de novo* assembled using the program Velvet 1.2.03 (Zerbino and Birney, 2008) with the following custom parameters: hash-length was 81–111 and coverage cut-off was 30. The draft genome assemblies were aligned in a pair-wise fashion using Mauve (Darling et al., 2004), and using *L. interrogans* serovar Icterohaemorrhagiae strain Lai as a reference. Details for the genomic assembly were summarized in **Table 1**. This genome shotgun project has been deposited at DDBJ/ENA/GenBank under the accession MSFX00000000. The version described in this paper is version MSFX01000000.

**Table 1:** Summary of *L. weilii* serovar Heyan strain L231 genome sequencing, assembly and annotation^∗^.

Strain name	L231
Contig number	208
N50	54,534 bp
Size of assembled genomes	4,268,389 bp
Maximum contig length	167,804 bp
Sequencing depth	118-folds
Assembly ratio	97.90%
Number of predicted genes	4,794
Gene average length	750 bp
GC content	40.67%
tRNA genes	37

*^∗^N50 is used to describe the length of contigs, and the size sum of contigs larger than N50 (54,534 bp) account for half of the genome size. Assembly ratio represents that 97.9% of all the sequenced reads was assembled into this genome (208 contigs).*

### Genome Analysis

Putative protein-coding sequences were predicted with the GeneMark program (Besemer et al., 2001) and functional annotation was performed by searching against the NCBI non-redundant protein database. Clusters of Orthologous Group (COGs) assignment (Tatusov et al., 2000) were conducted by RPS-BLAST using the NCBI CDD library (Marchler-Bauer et al., 2007). The metabolic pathways were constructed based on Kyoto Encyclopedia of Genes and Genomes (KEGG) database (Kanehisa et al., 2004). To further understand the evolution and pathogenesis of *L. weilii*, previously published genomes from several spirochetal species were also included in the phylogenetic analysis, such as those of non-pathogenic (*L. biflexa*), intermediate (*L. licerasiae*), and other pathogenic *Leptospira* species (Ren et al., 2003; Nascimento et al., 2004; Bulach et al., 2006; Picardeau et al., 2008; Zhong et al., 2011; Ricaldi et al., 2012; Lehmann et al., 2013; Xu et al., 2016) (**Table 2**). Genome alignments were performed using Mauve (Darling et al., 2004), and Pan-genome analysis was conducted with PGAP 1.11 using the MultiParanoid method (Zhao et al., 2012) with the following parameters: intraspecies coverage, 50%; intraspecies identity, 50%; interspecies coverage 50%; and interspecies identity 20%. A preliminary phylogenetic tree was constructed using PHYML (Guindon and Gascuel, 2003) and the concatenated protein sequences of 1359 putative orthologs selected by sequence similarity (Supplementary Table S1). To avoid errors derived from concatenating genes with different evolutionary pressures, we further selected genes from 235 ortholog groups that had been validated as having a neutral or nearly neutral evolution (Llanes et al., 2016). An alignment of the concatenated amino acid sequence of those genes was used to build a refined maximum likelihood tree for further investigating the evolutionary position of *L. weilii* serogroup Manhao.

**Table 2:** *Leptospira* strain included in the comparative genomic analysis.

Strain name	Species	Serovar	Genebank accession.	Lifestyle
56105	*L. weilii*	Sarmin	SRX673804	Pathogenic
56145	*L. weilii*	Worsfoldi	SRX673805	Pathogenic
56621	*L. weilii*	Anhoa	SRX673806	Pathogenic
56622	*L. weilii*	Hainan	SRX673807	Pathogenic
56646	*L. weilii*	Whitcombi	SRX673808	Pathogenic
56655	*L. weilii*	Mini	SRX673809	Pathogenic
56674	*L. weilii*	Menglian	SRX673811	Pathogenic
56679	*L. weilii*	Liangshan	SRX673810	Pathogenic
56601	*L. interrogans*	Icterohaemorrhagiae	NC_004342.2, NC_004343.2	Pathogenic
L550	*L. borgpetersenii*	Hardjobovis	NC_008508.1, NC_008509.1	Pathogenic
3522 CT	*L. kirschneri*	Cynopteri	AHMN00000000.2	Pathogenic
1342KT	*L. santarosai*	Shermani	AOHB00000000.2	Pathogenic
CZ 214T	*L. noguchii*	Panama	AKWY00000000.2	Pathogenic
L 60^T^	*L. alexanderi*	Manhao	AHMT00000000.2	Pathogenic
56643	*L. alexanderi*	Weaveri	SRX673674	Pathogenic
56650	*L. alexanderi*	Nanla	SRX673672	Pathogenic
56159	*L. alexanderi*	Erinacei auriti	SRX672003	Pathogenic
56640	*L. alexanderi*	Banna	SRX673675	Pathogenic
56659	*L. alexanderi*	Lichuan	SRX673676	Pathogenic
80–412	*L. alstonii*	Pingchang	AOHD00000000.2	Pathogenic
Bejo-Iso9T	*L. kmetyi*	Malaysia	AHMP00000000.2	Pathogenic
Ames	*L. biflexa*	Patoc	NC_010842.1, NC_010845.1,NC_010846.1	Non-pathogenic
VAR 010	*L. licerasiae*	Varillal	NZ_AHOO00000000.2	Intermediate

## Results and Discussion

### Virulence Analysis

**FIGURE 1:**
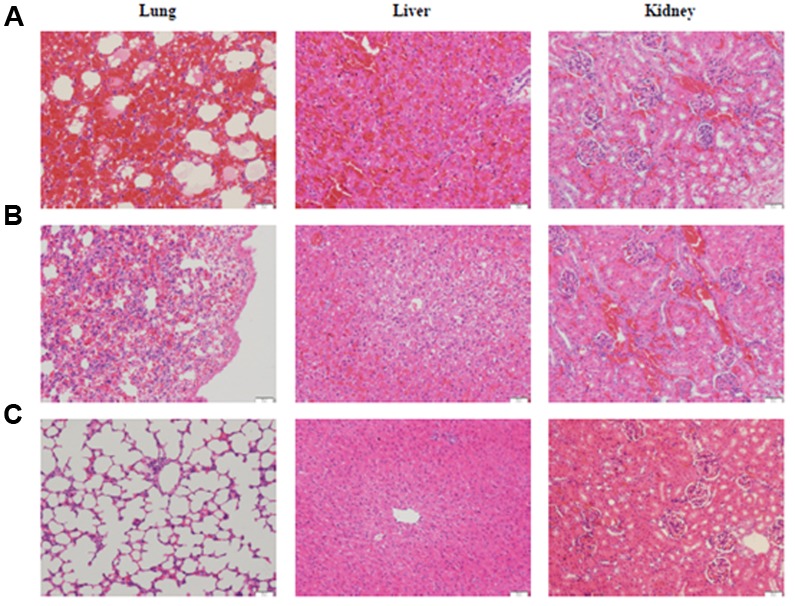
**Histological sections in the lung, liver and kidney of guinea pigs infected with *L. interrogans* serovar Lai strain Lai (A)**, *L. weilii* serovar Heyan strain L231 **(B)** and culture media **(C)**. HE staining, each bar indicates 100 μm.

To evaluate the virulence of the *Leptospira* isolates, the guinea pig model of leptospirosis were used in this study (Merien et al., 1998; Nally et al., 2004). The results showed that distinct clinical symptoms such as decreased activity ruffled hair coat and hemorrhage from the mouth in some animals were observed on day 4 post-infection from challenged group. No abnormalities were found in the negative control guinea pigs. While most of challenged-animals appeared moribund on day 7 post-infection, guinea pigs from all groups were sacrificed at day 7. Post-mortem analysis showed multiple areas of hemorrhaging visible on the surfaces of the lungs and extensive hemorrhaging on peritoneal surfaces (data not shown). The histopathological analysis further demonstrated that these infected animals developed typical leptospirosis pathological changes, including significant intra-alveolar hemorrhage, edema of alveolar septa and inflammatory infiltrate in the lungs, renal epithelium degeneration, interstitial nephritis, and glomerular capillary congestion in the kidneys, hepatic cell vacuolar degeneration and inflammatory infiltration (**Figure 1**).

It has been reported that *Leptospira* serogroups have specific host preferences; rats are the reservoir for serogroup Icterohaemorrhagiae while house mice for serogroup Ballum (Levett, 2001; Ko et al., 2009). Furthermore, previous studies also found that two strains of *L. borgpetersenii* serovar Hardjobovis have different capacities for infecting animal models in virulence studies, suggesting that the virulence of *Leptospira* spp. is strain dependent (Faine et al., 1999). *Leptospira* serogroup Manhao, is only found in China, which includes Lincang, Lushui, Qingshui, Manhao, Lichuan, and Heyan serovars. Until now, all isolates of serogroup Manhao except only one (porcine) were from human patients (Qin et al., 2004). Animal experiments showed that human isolate L231 of *Leptospira* serovar Heyan was a low virulence strain in guinea pigs requiring a high dosage of inoculation (1 × 10^9^) to induce mortality, and is able to infect rodents, suggesting that some rodent species may serve as animal reservoirs of serogroup Manhao in China. Serogroup Icterohaemorrhagiae is the most predominant epidemic-causing serogroup (among 18) with more than 70 serovars (Zhang et al., 2015). Some serovars from this serogroup are only found in China (Qin et al., 2004; Zhang et al., 2012). To understand comprehensively the pathogenesis of leptospirosis and develop a better control strategy for this disease, further studies are required to investigate the epidemiology, maintenance host and the virulence mechanisms of the pathogenic *Leptospira* serogroups, especially for those found exclusively in China.

### DNA Sequencing and Preliminary Characterization of Genomic Features

*Leptospira* strain L231 was characterized by 16S rRNA gene sequencing. The amplification and sequencing of the 16S rRNA gene indicated that the strain L231 belonged to the pathogenic species *L. weilii*, which was different from other serovars of serogroup Manhao (i.e., species of serovars Manhao and Lichuan was *L. alexanderi*; Brenner et al., 1999; Fouts et al., 2016). LPS is the major antigen involved in *Leptospira* serological classification (Kmety and Dikken, 1993; de la Pena-Moctezuma et al., 2001), whereas species differentiation is closely associated with DNA heterogeneity (Brenner et al., 1999). Recently, it has been reported that bacterial strains within the same serovar grouping may belong to distinct species, having evolved through genetic drift, homologous recombination or horizontal gene transfer (de la Pena-Moctezuma et al., 1999; Xu et al., 2016).

**FIGURE 2:**
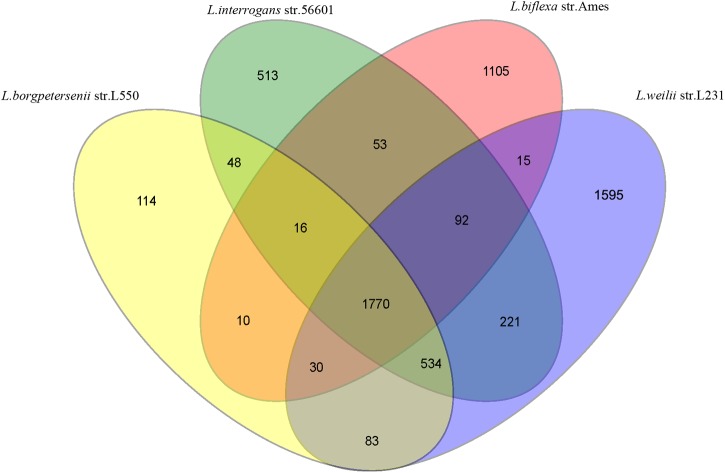
**Venn diagram showing numbers of unique and shared orthologs amongst *L. interrogans*, *L. borgpetersenii*, *L. biflexa*, and *L. weilii* strain L231**.

To further characterize the new serovar uncovered in this study, genome sequencing technique with high resolution was used. The draft genome was assembled into 208 contigs with a total length of 4,268,389 bp, 300 kb larger than the smallest *Leptospira* genome (*L. borgpetersenii* with an average genome size of 3.88 Mb). The genomic analysis showed that the G+C content was 40.67%. Gene prediction on the strain L231 genome showed that 84.2% of the total sequence comprises coding regions, containing 4,794 ORFs with an average size of 750 bp. Among all of the protein-coding genes (CDSs) in strain L231, 46.1% were assigned to a functional category of COGs (Supplementary Table S2). Further analysis showed that the L231 genome shares 2617 orthologs with *L. interrogans*, 2417 with *L. borgpetersenii* and 1907 with *L. biflexa*. A total of 1770 orthologs were present in all genomes analyzed (**Figure 2**). It was also determined that 1,595 ortholog clusters of strain L231 share no orthologs with the other three species, with 1,358 of these (78.4%) annotated as hypothetical proteins (data not shown).

Similar to other pathogenic and slow-growing species (Ren et al., 2003; Bulach et al., 2006), a total of 37 transfer RNA (tRNA) genes were revealed in strain L231 genome, representing all 20 amino acids. In contrast, the fast-growing *L. biflexa* has 35 tRNA genes (Picardeau et al., 2008). The finding further suggests that the growth rate of *Leptospira* is not restricted by low number of tRNA genes, but rather is associated with distinct metabolic capacities amongst *Leptospira* spp. (Ren et al., 2003; Picardeau et al., 2008). To further analyze other non-coding RNA elements in the strain L231 genome, riboswitch predictions were performed using Riboswitch Scanner (Mukherjee and Sengupta, 2016). In agreement with the previous studies (Ricaldi et al., 2012; Fouts et al., 2016), three riboswitches associated with transport and vitamin biosynthesis were found. Of these, two elements are located upstream of *L231_gene1384* (encoding a putative TonB-dependent receptor) and *L231_gene3285* (encoding a putative sirohydrochlorin cobaltochelatase), respectively. It is reported that a complete genetic support for vitamin B12 autotrophy exists in pathogenic but not saprophyte *Leptospira*, suggesting the synthesis of B12 *de novo* may be critical for *Leptospira* to infect mammals in the face of B12 sequestration by host (Fouts et al., 2016). Although the mechanistic details of these riboswitches are still unknown, these non-coding RNAs seems to be involved in the regulation of transport and biosynthesis of vitamin B12 in pathogenic *Leptospira*, facilitating adaptation to different host environments. Furthermore, another riboswitch, found in strain L231, is upstream of *L231_gene2603* (encoding a hydroxymethylpyrimidine phosphate synthase, ThiC), which exists in almost all reported leptospiral genomes.

### Energy Metabolism

Based on the KEGG analysis, the stain L231 had the potential to synthesize 17 amino acids; methionine, histidine and asparagine being the exceptions. Although some enzymes for *de novo* synthesis are not apparently present, methionine could be synthesized from homocysteine, histidine could be synthesized from an L-histidinol intermediate and isoleucine could be synthesized from 2-Oxobutanoate (Crout et al., 1980; Winkler and Ramos-Montañez, 2009; Ferla and Patrick, 2014). For the tricarboxylic acid (TCA) cycle, the gene for pyruvate carboxylase synthesis from pyruvate was not found in L231. Malate dehydrogenase (mdh; *L231_gene702*) and citrate synthase (*L231_gene1259*) were identified, the products of which catalyzed the synthesis of oxaloacetate from malate and citrate (Panchenko and Vinogradov, 1991).

Similar to other pathogenic *Leptospira* species (Ren et al., 2003; Nascimento et al., 2004), genes involved in the bacterial glycerol metabolism were found in the strain L231 genome. Those genes encode glycerol-3-phosphate dehydrogenase (*L231_gene224*), glycerol-3-phosphate transporter (*L231_gene320*), glycerol kinase (*L231_gene721*), and 1-acyl-sn-glycerol-3-phosphate acyltransferase (*L231_gene79*). These findings suggest that that glycerol and fatty acids may be generated from phospholipid degradation (Nascimento et al., 2004). It is well known that glycerol is the central structural component of the major classes of biological lipids, triglycerides and phosphatidyl-rich phospholipids, and also serves as an important intermediate in carbohydrate and lipid metabolism (Thomas et al., 1990).

### LPS Biosynthetic System

Many studies have demonstrated that leptospiral LPS plays an important role on host immunity responses and in serovar determination in *Leptospira* (Kmety and Dikken, 1993; de la Pena-Moctezuma et al., 1999, 2001; Faine et al., 1999). Changes in genes of the LPS biosynthetic pathway (*rfb*) locus are associated with serovar diversity among *Leptospira* spp. (de la Pena-Moctezuma et al., 1999, 2001).

**FIGURE 3:**
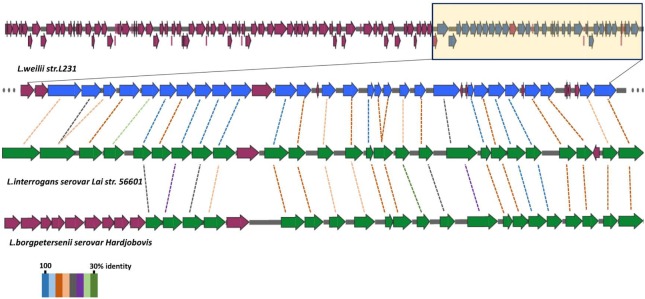
**Structure of *Leptospira rfb* locus gene clusters.** The *rfb* region of *L. weilii* serovar Heyan strain L231 are compared with the homology in *L. interrogans* serovar Lai (Ren et al., 2003) and *L. borgpetersenii* serovar Hardjobovis (Kalambaheti et al., 1999). The CDSs marked with yellow in *L. weilii* strain L231 *rfb* region are homologous to other two species. BLASTP matches between CDSs are indicated by colored protein percent identity figures (see key).

In agreement with the previous findings (Fouts et al., 2016), a larger *rfb* locus (spanning approximately 111 kb was observed in the strain L231 genome (Supplementary Table S3), when compared with those of *L. interrogans* serovar Lai (36.0 kb) and *L. borgpetersenii* serovar Hardjobovis (36.7 kb; Mitchison et al., 1997; Kalambaheti et al., 1999; Ren et al., 2003). While loci from *L. interrogans* and *L. borgpetersenii*, respectively, contain 91 and 76 genes, strain L231 *rfb* locus contains 120 genes (**Figure 3**, Supplementary Figure S1 and Supplementary Table S3). Comparison of the nucleotide sequence of the *rfb* locus of serogroup Manhao serovar Manhao strain L60^T^ (121 kb) and Heyan strain L231 revealed more than 91.5% identity in the 73 kb homologous regions, including 81 pairs of orthologous genes (**Figure 3** and Supplementary Figure S1), hence reflecting the antigenic/serologic specificity of serogroup Manhao. Although most of the unique genes of *rfb* locus in strain L231 were hypothetical proteins, three genes encoding aminotransferase PglE were found (Supplementary Table S3). PglE belongs to the aspartate aminotransferase superfamily, and is involved in a novel sugar N,N′-diacetylbacillosamine biosynthesis, which contributes to bacterial membrane protein glycosylation (Riegert et al., 2015). Furthermore, those differences may occur in different serovars from the same serogroup, providing further evidence that different *Leptospira* genospecies or strains may be classified as being in the same serogroup or serovar (de la Pena-Moctezuma et al., 1999). Similar to other pathogenic *Leptospira* species, a complete dTDP-rhamnose biosynthesis gene cluster encoding *rfb ABCD* was present in strain L231 genome (*L231_gene1714*-*L231_gene1717*). Previous studies demonstrate that LPS of pathogenic *L. interrogans* contain high amounts of rhamnose (Vinh et al., 1989) whereas LPS of intermediately pathogenic *L. licerasiae*, occasionally causing human infection, has a small amount of rhamnose (Patra et al., 2015). Furthermore, compared with those of intermediately pathogenic *Leptospira*, the chemical composition of pathogenic *L. interrogans* serovar Copenhageni LPS comprises of some specific sugars, such as fucose and acetylglucosamine (Patra et al., 2015). It is well known that LPS is involved in pathogenesis of Gram-negative bacteria, including pathogenic *Leptospira* species. Composition and structural complexity of LPS residues in pathogenic *Leptospira* species are thought to be greater than those of intermediate and non-pathogenic species (Murray et al., 2010; Patra et al., 2015). Due to large variations in *rfb* gene clusters among different pathogenic species, more experimental investigations are needed to comparatively characterize the LPS of other pathogenic species, particularly those species with larger *rfb* gene cluster, to better understand mechanisms of pathogenesis and colonization of pathogenic leptospires.

### Cell Motility, Putative Virulence Factors and Protein Secretion

Bacterial motility has long been shown to be a central factor in enabling pathogens to penetrate host tissue barriers during infection, and it has also been suggested that it plays a crucial role in the pathogenesis of *Leptospira* (Liao et al., 2009). We identified 32 CDSs encoding motility-related proteins. Most CDSs (22/32) encoded putative proteins involved in flagellar biosynthesis and export, while the remaining CDSs (10/32) were predicted to encode proteins from bacterial chemotaxis.

Potential virulence factors in the strain L231 genome were identified using BLAST search in the VFDB database. A total of 407 putative virulence factors are presented in Supplementary Table S4. Some of these proteins (such as hemolysins and flagella) have been demonstrated to be virulence factors in pathogenic *Leptospira* species, although most are homologous to proteins that had been shown to be virulent in other pathogens. Furthermore, previous studies report that some lipoproteins may be essential for leptospiral virulence, which play important role in infection (Koizumi and Watanabe, 2003; Ristow et al., 2007). We also identified 48 putative lipoproteins and three OmpA homologs (Supplementary Table S5). It is suggested that further experiments should be conducted to investigate the function of potential virulence in *L. weilii* strain L231 infection.

Currently, at least eight different pathways have been reported in Gram-negative bacteria for secretion of proteins across the cell envelope (Pallen et al., 2003; Kostakioti et al., 2005). Eleven genes, separately involved in Type II and the general secretory (Sec)-signal recognition particle (SRP) systems, were revealed in the genome of strain L231. The Type II secretion genes are organized in a single cluster (*L231_gene1861-1865*) in strain L231. This translocation system is Sec-dependent, and is responsible for the extracellular transport of a large number of toxins and hydrolytic enzymes (Kostakioti et al., 2005; Haake and Zuckert, 2015). These results suggested that strain L231 utilize Type II secretory systems to export proteins from the cytoplasm. Furthermore, we identified 26 genes involved in ATP-binding cassette (ABC) transporters, but only four sets of complete ABC type clusters for transporting lipopolysaccharides, lipoproteins and sulfates.

### Phages and CRISPR-Cas System

Prophages are commonly found in bacterial genomes, where they play an important role in the evolution of the host bacteria (Fortier and Sekulovic, 2013). Phage_Finder predictive analysis revealed one prophage (27,454 bp) in strain L231 genome (Supplementary Table S6), which have been also found in the genomes of other pathogenic, or intermediate *Leptospira* species (Fouts et al., 2016). In order to be able to survive in the host, intermediate and pathogenic *Leptospira* species have to adapt to a variety of growth environments and conditions (Xu et al., 2016). It is speculated that the resistance mechanisms and metabolic advantages provided by prophages facilitate pathogen adaptation and evolution into a variety of different genospecies, allowing colonization of a wide variety of different mammalian hosts.

Clustered regularly interspaced short palindromic repeats (CRISPR) and CRISPR-associated (Cas) genes are found in many sequenced bacterial genomes, which contribute to resistance to exogenous genetic elements, such as plasmids and phages, as well as regulation of gene expression (Louwen et al., 2014). In the genome of strain L231, three CRISPRs with length of 756, 1,123 and 333 bp were revealed (Supplementary Table S7). Previous studies have shown that all nine pathogenic *Leptospira* species and some intermediate species have potential CRISPRs-Cas systems, whereas non-pathogenic species seem to lack these sequences, suggesting that specific CRISPRs-Cas systems among pathogenic species may play a role in invasion and evasion of host immune systems (Fouts et al., 2016). Furthermore, it was reported that pathogen subspecies of bacteria with variant CRISPR-Cas systems show substantial differences with respect to virulence (Louwen et al., 2014). Therefore, further studies aimed at the characterization of CRISPRs-Cas systems should be performed among pathogenic *Leptospira* spp., which will certainly provide deeper insight into the mechanisms leading to control of virulence via CRISPRs-Cas system.

### Comparative and Phylogenomic Analysis

Genomic analysis showed that the genome sequence similarity varied from less than 1% to more than 77% for pair-wise comparisons of strain L231 and other pathogenic, intermediate, and non-pathogenic species (**Table 3**). Furthermore, greater variation on predicted protein levels was found among different pathogenic *Leptospira* species. For example, the similarity between predicted homologs at the amino acid level in the *L. weilii* strain L231 genome was 85.1 and 92.3% in comparison with *L. noguchii* and *L. borgpetersenii*, respectively (**Table 3**). These observations further suggested high genomic plasticity among pathogenic *Leptospira* spp. (Xu et al., 2016).

**Table 3:** Comparison of *L. weilii* serovar Heyan strain L231 with other *Leptospira* species^∗^.

Species	Genomic similarity	Gene identity	Protein identity
*L. interrogans*	43.20%	82.6% (2507)	84.7% (2618)
*L. borgpetersenii*	61.50%	91.9% (2465)	92.3% (2550)
*L. kirschneri*	43.30%	82.9% (2490)	84.9% (2581)
*L. alexanderi*	77.10%	95.3% (2699)	95.8% (2701)
*L. noguchii*	42.30%	82.7% (2508)	85.1% (2603)
*L. santarosai*	59.30%	89.2% (2587)	90.6% (2603)
*L. alstonii*	57.40%	85.5% (2611)	87.2% (2648)
*L. kmetyi*	40.50%	83.5% (2461)	85.1% (2584)
*L. biflexa*	0.12%	81.6% (213)	51.4% (2119)
*L. licerasiae*	1.10%	80.9% (764)	62.9% (2344)

*^∗^Number in brackets represented number of gene or protein orthologs.*

To further understand the molecular evolution of *L. weilii*, the pan-genome for *L. weilii* species was calculated based on nine available genomes. Accumulation curve for the pan-genome suggested it to be open, with a γ = 0.3486. According to Heap’s Law, the pan-genome is considered open when the power law exponents γ > 0 (Supplementary Figure S2). The result revealed that the size of the *L. weilii* pan-genome is relatively large, comprising 9,639 genes. The large pan-genome in *L. weilii* species may thus provide more insights into bacterial evolution because pathogenic *Leptospira* species adapt to distinct environments via multiple ways of exchanging genetic material (Bulach et al., 2006). Further analysis identified 2,771 annotated core genes in *L. weilii* species (Supplementary Table S8), accounting for only 28.7% of total pan-genes. In agreement with our previous studies (Xu et al., 2016), some known virulence factors including bacterial toxins and surface proteins were found in the core genomes of *L. weilii* species. In addition, many of the core genes were also involved in metabolic pathways, together reflecting that conserved core genes may be closely related to bacterial pathogenesis.

**FIGURE 4:**
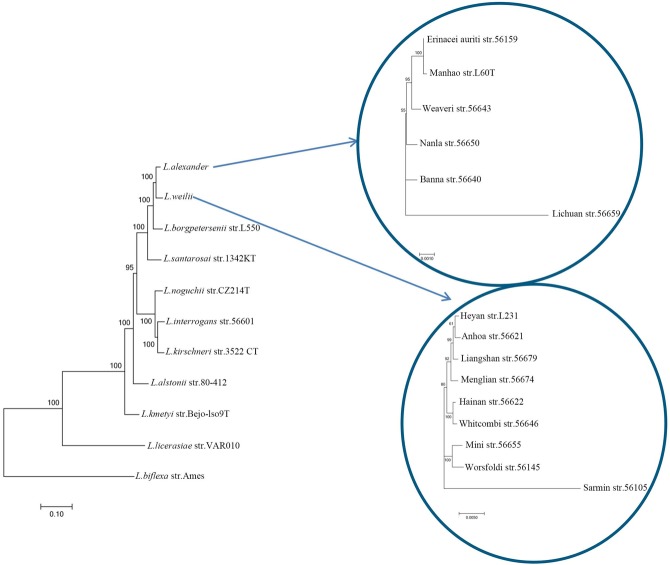
**Phylogenetic analysis of *Leptospira* species.** The tree was constructed using the concatenated orthologous proteins of each strain. Scale bar indicated an evolutionary distance of 0.1 amino acid substitutions per position for phylogenetic tree of *Leptospira* species, 0.001 amino acid substitutions per position for tree of *L. alexanderi* and 0.005 amino acid substitutions per position for tree of *L. weilii*. Bootstrap values are shown for branches separating different species or serovars.

A preliminary phylogenetic tree built with concatenated sequences of putative orthologs (Supplementary Figure S3), shows that pathogenic, intermediate and non-pathogenic species are separately clustered, which is consistent with previous phylogenetic hypotheses based on 16S rRNA gene sequence analysis (Brenner et al., 1999). Different from the cluster formed by those closely related *L. interrogans*, *L. kirschneri* and *L. noguchii*, *L. weilii* seems to be closer to *L. borgpetersenii* and *L. alexanderi*. Previous studies have reported that neutral synonymous sites from genes have a reasonably better phylogenetic signal (Massey et al., 2008). To further explore the origin of *L. weilii* serogroup Manhao and its evolutionary relationship with other member of the genus *Leptospira*, a set of 235 ortholog groups predicted to be subjected to neutral evolution (with an overall dN/dS ratio between 0.2–2.0; Llanes et al., 2016) were used to reconstruct a refined phylogenetic tree (**Figure 4**). The topology of this new tree is in agreement with that of Supplementary Figure S3, but individual *L. weilii* and *L. alexanderi* specimens can now be subdivided. The phylogenetic tree shows three separate subgroups within the *L. weilii* clade. The evolutionary position of serovar Heyan (strain L231) is closer to serovar Anhoa (strain 56621). The topology also revealed that other serovars from serogroup Manhao, namely serovar Lichuan (strain 56659) and serovar Manhao (strain L60^T^), belong to *L. alexanderi* rather than to *L. weilii*. These findings further support the hypothesis that the presence of similar *rfb* loci in different *Leptospira* species may be closely associated with gene acquisition via lateral gene transfer (Llanes et al., 2016).

## Conclusion

In this study, our experiment evidences indicated a new serovar Heyan strain L231 can infect rodents, suggesting that the monitoring of specific host reservoir of serogroup Manhao involved in transmission should be strengthened for better control of leptospirosis in China. Genomic analysis provided comprehensive information about strain L231 molecular features. Distinctive LPS biosynthesis locus of serovar Heyan provides further evidence for the unique antigenic/serologic specificity of serogroup Manhao. More importantly, our comparative genomic analysis confirmed a high genomic plasticity among pathogenic species, indicating that different species may undergo a distinct evolutionary process to adapt to different hosts or environmental niches. Unlike the other two serovars of serogroup Manhao, serovar Heyan seems to be more closely related to serovar Anhoa, within the *L. weilii* clade. All these findings have important implications for understanding evolution of pathogenic *Leptospira* in China.

## Author Contributions

JW and YX conceived and designed the study. YX, YZ, JZ, ZL, SC, XX, and QY performed the experiments. HZ, YW, Y-FC, and YX conducted the bioinformatic analyses of the data. JW, YX, Y-FC, and HZ wrote the paper. All authors read and approved the final manuscript.

## Conflict of Interest Statement

The authors declare that the research was conducted in the absence of any commercial or financial relationships that could be construed as a potential conflict of interest.

## Footnotes

1 http://www.ncbi.nlm.nih.gov/BLAST
