# Supplementary material for: Genomic Analysis of a New Serovar of Leptospira weilii Serogroup Manhao
Source: Front Microbiol. 2017 Feb 2;8:149. doi: 10.3389/fmicb.2017.00149 (PMC5288384; doi:10.3389/fmicb.2017.00149)
Supplement: Supplementary file 2 [file Data_Sheet_2.pdf]

Supplementary Material

Genomic analysis of a new serovar of *Leptospira weilii* serogroup Manhao

Yinghua Xu[1]#, Huajun Zheng[2,3]#, Ying Zhang[1]#, Yuezhu Wang[3], Jinlong Zhang[1], Zhe Li[1], Shenghui Cui[1], Xiaofang Xin[1],Qiang Ye[1], Yung-Fu Chang[4], Junzhi Wang[1]*

***Corresponding author:** Dr. Junzhi Wang, E-mail: wangjz@nifdc.org.cn;

#These authors contributed equal to this work

1 Supplementary Figures

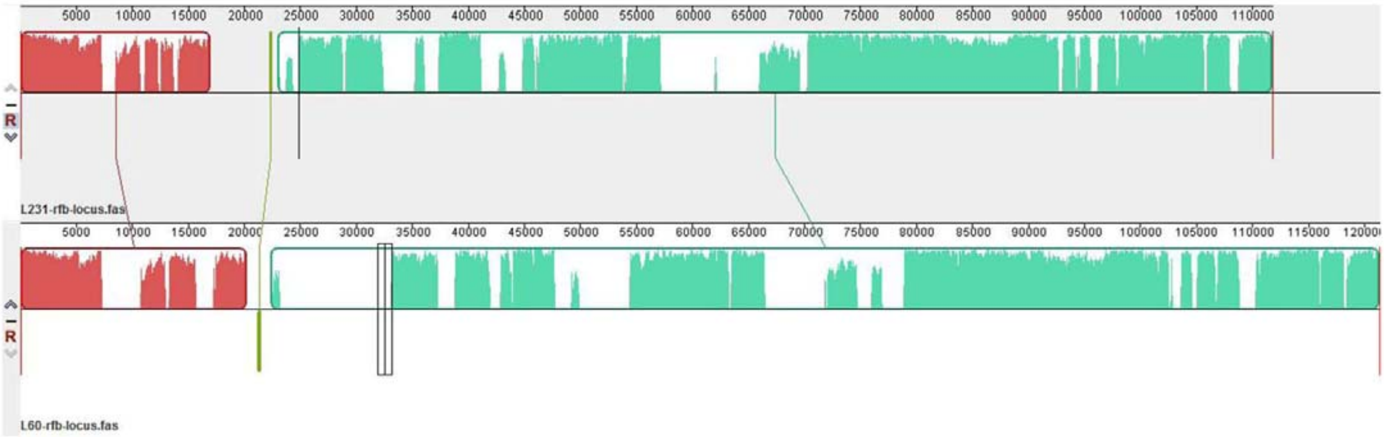

Supplementary Figure 1. *Leptospira rfb* locus gene cluster alignment. The alignment was performed using Mauve and showed a linear organization of *rfb* locus gene clusters of serogroup Manhao serovars Heyan strain L231 and Manhao strain L60^{T}. The horizontal bars represent gene cluster size (bp).

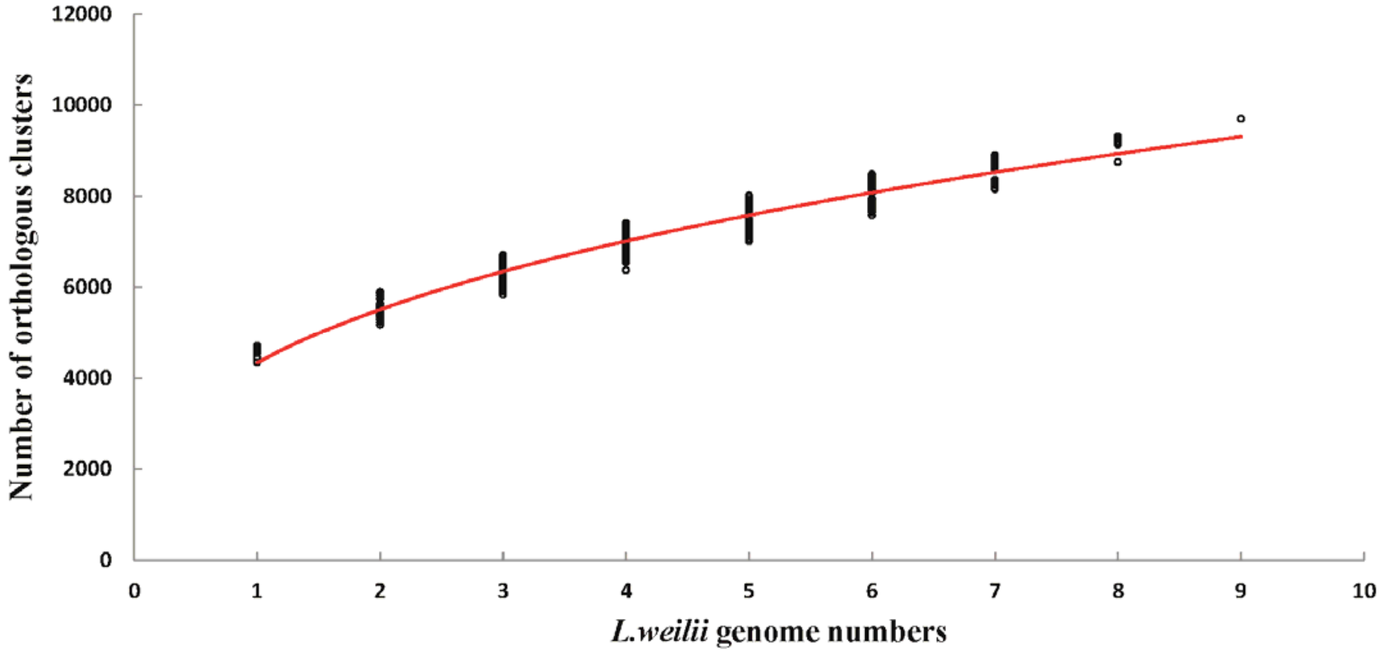

Supplementary Figure 2. Accumulation curves for the pan-genome of pathogenic *L.weilii* species. Circles represented number of ortholog clusters for each genome. The red curve was a least squares fit of the power law $y = k\ x^{\gamma}$ to medians, with the exponent $\gamma>0$ indicating an open pan-genome.

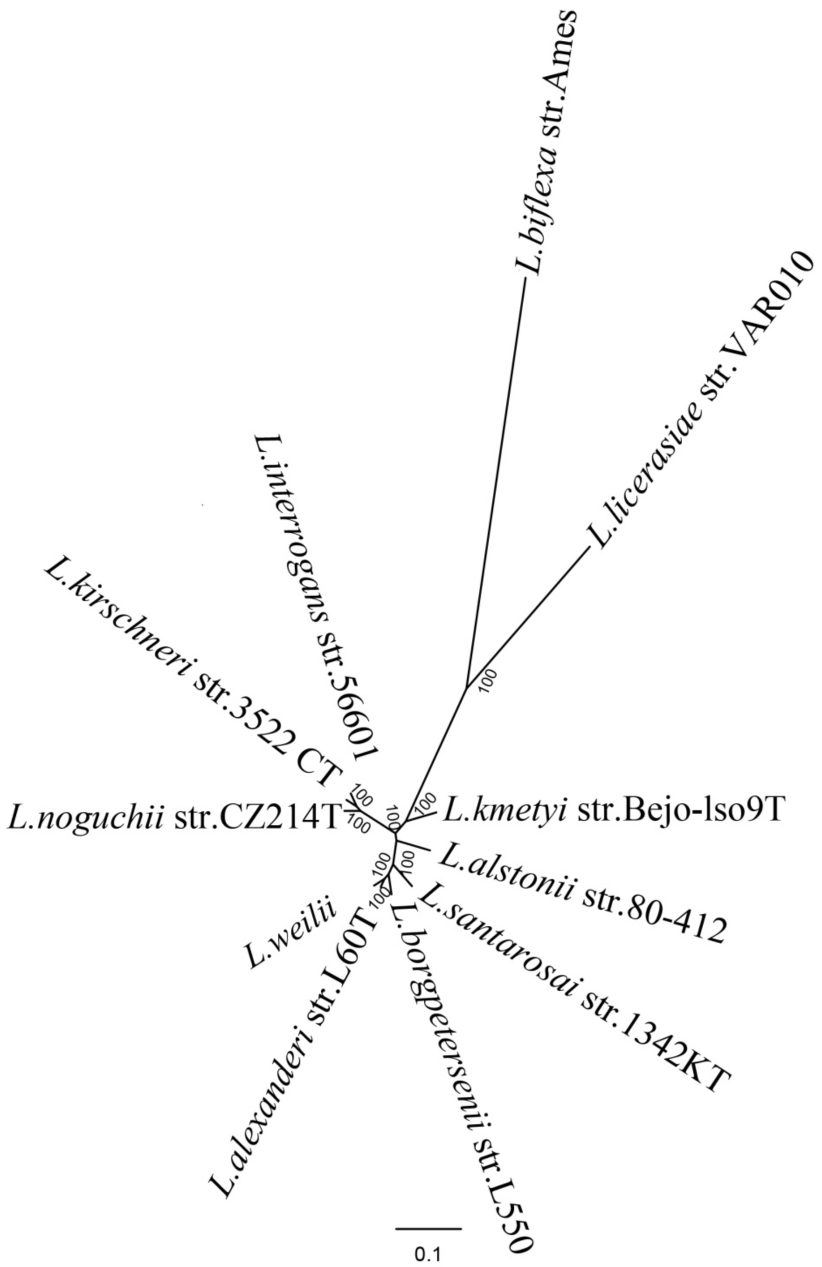

Supplementary Figure 3. Phylogenetic analysis of *Leptospira* species. The tree was constructed using the concatenated orthologous proteins of each strain. Scale bar indicated an evolutionary distance of 0.1 amino acid substitutions per position. Bootstrap values are shown for branches separating different species.

2 Supplementary Tables

Supplementary Table S1. Ortholog groups used in genomic analysis for *Leptospira* species.

Supplementary Table S2. Distribution of general protein functions based on the COG function classification scheme

Supplementary Table S3. Genes involved in lipopolysaccharide synthesis in the genome of *L. weilii* serovar Heyan strain L231

Supplementary Table S4. Predicted virulence factor in *L. weilii* serovar Heyan strain L231

Supplementary Table S5. Putative lipoproteins in *L. weilii* serovar Heyan strain L231

Supplementary Table S6. Predicted prophage in the genome of *L. weilii* serovar Heyan strain L231

Supplementary Table S7. CRISPRs in the genome of *L. weilii* serovar Heyan strain L231

Supplementary Table S8. Core genome of *L. weilii* species
